# Supplementary material for: Microbial Diversity in Subarctic Biocrusts from West Iceland following an Elevation Gradient
Source: Microorganisms. 2021 Oct 21;9(11):2195. doi: 10.3390/microorganisms9112195 (PMC8624075; doi:10.3390/microorganisms9112195)
Supplement: Supplementary file 1 [file microorganisms-09-02195-s001.zip › microorganisms-1380989-supplementary.pdf]

Supplementary Table S1. List of primers used for the (a) amplicon and (b) sanger sequencing.

a.

Gene	Organism	Primers	Sequence	Primer reference
16S	Bacteria	341F	CCTACGGGRSGCAGCAG	
		802R	TACNVGGGTATCTAATCC	
	Cyanobacteria	CYA_F	GGGGAATYTTCCGCAATGGG	Nübel et al. 1997
		CYA_R	GACTACWGGGGTATCTAATCCCWTT	
ITS	Fungi	Fungi_F	CAHCGATGAAGAACGYRG	Frey et al. 2016
		Fungi_R	TCCTSCGCTTATTGATATGC	
18S	Eukaryotes	tarEuk_F	CCAGCASCYGCGGTAATTCC	Stoeck et al. 2010
		tarEUk_R	ACTTTCGTTCTTGATYRA	

b.

Organisms	Molecular analysis	Primers	Sequence	Primer reference
Cyanobacteria	PCR, sequencing	BS-1F	GATCCTKGCTCAGGATKAACGCTGGC	Marin et al. 2002
	sequencing	BS3F	GCCAGCAGCCGCGGTAA	Wilmotte et al. 1993
	sequencing	BS4F	GYAACGAGCGCAACCC	Wilmotte et al. 1993
	sequencing	BS8R	AAGGAGGTGATCCAGCCGCA	Wilmotte et al. 1993
	sequencing	BL9R	TTTGCGGCCGCTCTGTGTGCCTAGGTATCC	Marin et al. 2002
	PCR	CPL-10R	GCCGGCTCATTCTTCAAC	

Eukaryotic microalgae	PCR, sequencing	EAF3	TCGACAATCTGGTTGATCCTGCCAG	Marin 2003
	sequencing	E528	TGCCAGCAGCYGCGGTAATTCCAGC	Marin 1998
	sequencing	536R	GWATTACCGCGGCKGCTG	Marin 1998
	sequencing	BR	TTGATCCTTCTGCAGGTTCACCTAC	Marin 1998
	PCR	ITS055R	CTCCTTGGTCCGTGTTTCAAGACGGG	Marin 1998

Supplementary Table S2. Differences of microbial community composition between the five studied sites based on pairwise PERMANOVA (9999 permutations). Asterisks corresponds to the level of significance (*p<0.05, **p<0.01, ***p<0.001).

**Pairs**	**Bacteria**		**Cyanobacteria**		**Fungi**		**Eukaryotes**	
	**r^2**	***F***	**r^2**	***F***	**r^2**	***F***	**r^2**	***F***
KRA vs. BOR	0.34	3.04*	0.51	5.24*	0.27	2.27*	0.50	5.02*
KRA vs. FIF	0.21	1.55^{ns}	0.24	1.55^{ns}	0.18	1.36^{ns}	0.29	2.06*
KRA vs. LSK	0.27	2.18*	0.28	1.92*	0.26	2.07*	0.41	2.73^{ns}
KRA vs. GIL	0.29	2.43*	0.30	2.09^{ns}	0.30	2.61*	0.32	1.91^{ns}
BOR vs. FIF	0.39	3.80*	0.55	7.33*	0.32	2.89*	0.44	4.62*
BOR vs. LSK	0.53	6.89*	0.71	14.78^{ns}	0.37	3.49*	0.65	9.34*
BOR vs. GIL	0.53	6.85*	0.86	36.42*	0.43	4.52*	0.59	7.07*
FIF vs. LSK	0.21	1.59^{ns}	0.27	2.25*	0.27	2.23*	0.34	2.53*
FIF vs. GIL	0.35	3.24*	0.42	4.35*	0.28	2.32*	0.30	2.17*
LSK vs. GIL	0.41	4.11*	0.40	4.02*	0.37	3.58*	0.37	2.37^{ns}

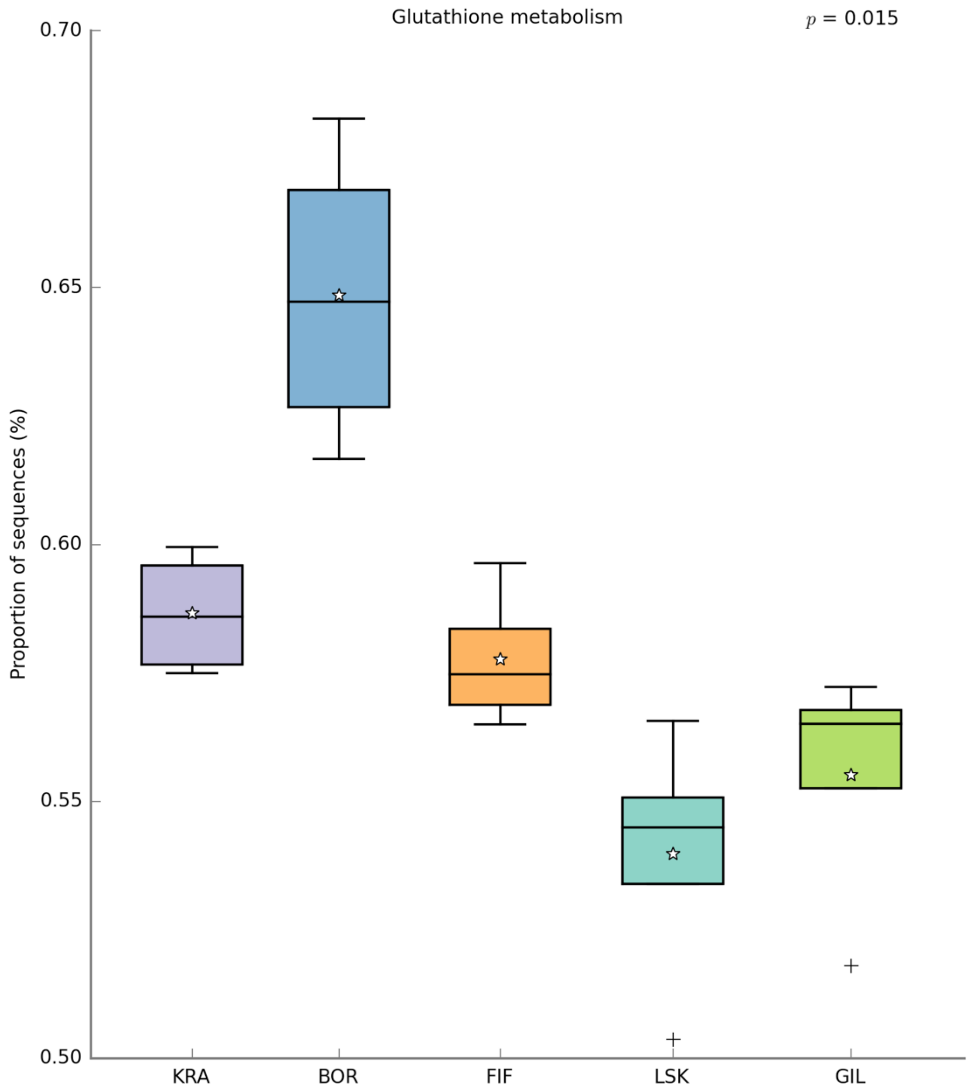

Supplementary Figure S1 – Proportion of bacterial sequences potentially involved in glutathione metabolism estimated by matching of 16S rRNA gene amplicons with genomes from reference database (KEGG). The sites are listed in order of altitudinal gradient (10-157 m a.s.l.): KRA – Krákunes, BOR – Borgarfjarðarbraut, FIF – Fiflholt, LSK – Litla Skard, GIL – Giljar.

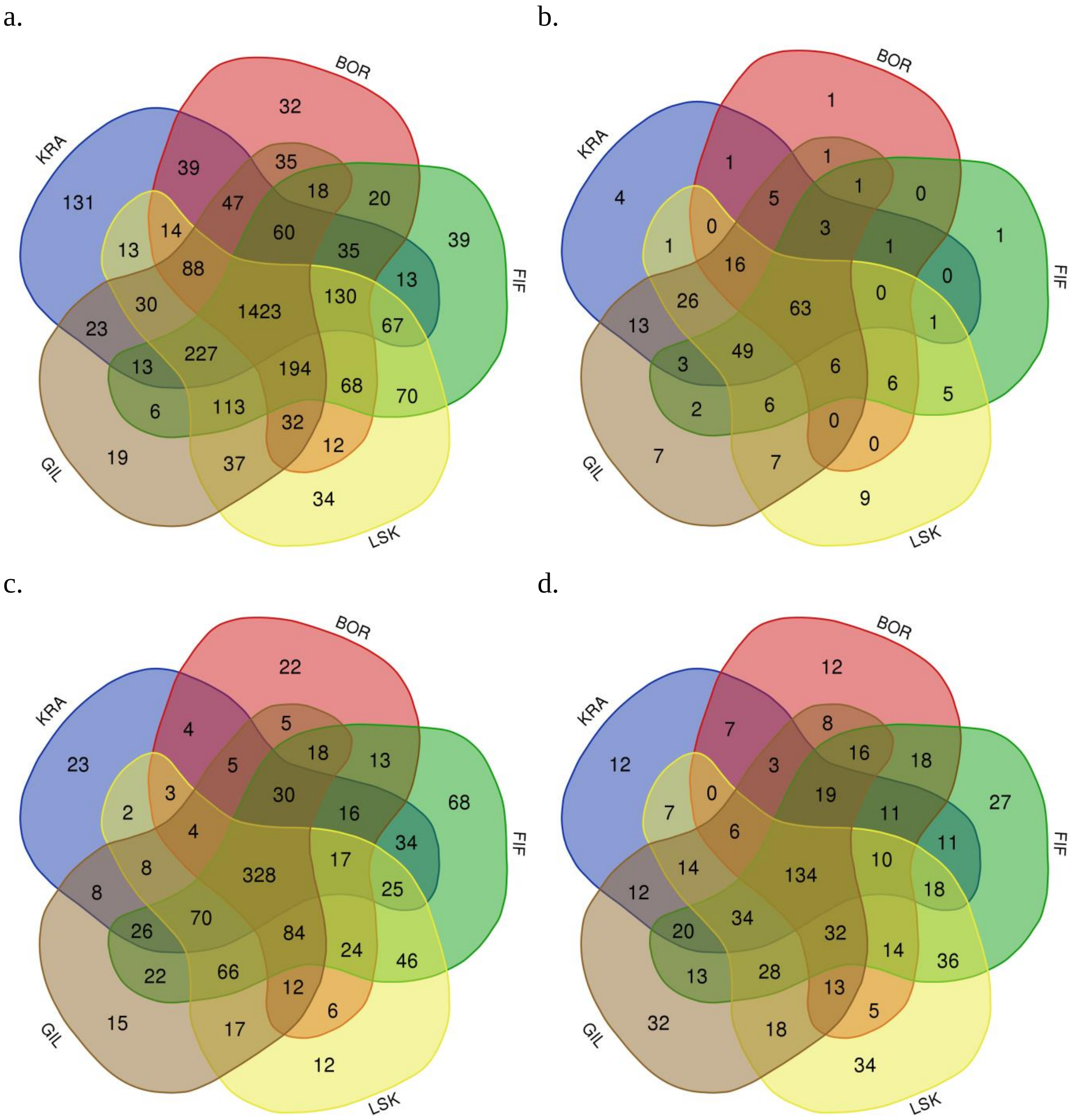

Supplementary Figure S2 – Venn diagram showing the relationships between the studied sites based on shared OTUs of bacteria (a), cyanobacteria (b), fungi (c) and eukaryotes (d).
